# Further evidence for embodied cognition: The link between spontaneous respiratory oscillations and number processing

**DOI:** 10.1371/journal.pone.0351764

**Published:** 2026-06-26

**Authors:** Sebastiano Cinetto, Hurcan Andrei Senyuva, Marco Zorzi, Mariagrazia Ranzini

**Affiliations:** 1 Padova Neuroscience Center, University of Padova, Padova, Italy; 2 Department of General Psychology, University of Padova, Padova, Italy; University of Hamburg, GERMANY

## Abstract

Embodied accounts of cognition propose that bodily states shape mental representations. We test this proposal in numerical cognition by asking whether spontaneous respiration biases random number generation (RNG). Building on prior work by Belli and collaborators (2021), we adopt a self-paced handwriting RNG paradigm that preserves the continuous, cyclic nature of breathing. Healthy adults will write digits 1–9 on a tablet while abdominal respiration is recorded via a piezoelectric belt. For each trial, we will extract the breathing phase (inhalation/exhalation) and the abdominal expansion at that moment. We preregister three hypotheses: (Hp1) inhalation will bias production toward larger numbers relative to exhalation; (Hp2) greater abdominal expansion will predict larger numbers independent of phase; and (Hp3) inhalation and larger expansion will be associated with increased written-digit size. The predefined sample size (N = 30) will provide 99.9% power to detect effects on the order reported by prior work. By coupling fine-grained respiratory dynamics with self-paced behavior, the study offers a stringent test of whether interoceptive and somatosensory signals couple with abstract magnitude processing. Confirmatory evidence would provide convergent, method-robust support for embodied influences on numerical cognition and will reveal a parallel impact on action, strengthening brain–body accounts of magnitude representation.

## Introduction

The nuanced connection between brain and body and the relevance of bodily functions for understanding mental processes and representations has been extensively stressed by the embodied cognition literature [1–3]. In the last decades a large amount of empirical evidence has highlighted the key role of sensorimotor experiences in shaping cognitive functions, such as language [2,4] or numerical cognition [5–7], thereby supporting the hypothesis that cognition is indeed ‘embodied’.

It has been proposed that the influence of sensorimotor experiences on cognitive processing can be referred to *grounded* (universal), *embodied* (learning-related) and *situated* (task-dependent) aspects [8]. First, sensorimotor mechanisms shape cognition when interaction with the environment causes experience of universal laws, such as gravity, or direction of growth; this process is known as grounding. Second, the occurrence of repetitive motor actions during cognitive processing causes emergence of long-lasting associations, such as culture-specific habits that define the direction of reading and writing; this process is known as embodiment. Third, specific environmental characteristics or current states cause contingent sensorimotor experience influencing behavior; this process concerns situated aspects of cognition. In everyday life activities, grounded, embodied, and situated aspects of cognition are usually intermingled [8].

The link between sensorimotor experience and cognition is well illustrated by numerical cognition research [6]. Numerical cognition concerns the study of the mental and neural processes involved in dealing with numbers and quantities [9]. A large body of evidence indicates that the processing of numerical magnitude is influenced by grounded, embodied, and situated aspects. For instance, it has been shown that number magnitude has an impact on hand grasping actions [7,10]. These findings are in line with the ATOM proposal (A Theory Of Magnitude [11]), which hypothesizes a unitary system for magnitude representation in the parietal cortex integrating modality-specific quantity-related signals. The idea that the interplay between number and hand action might result from neuroanatomical constraints is also supported by the extensive overlap between number, reach, and grasp neural networks [7].

Cognition is also influenced by interoception which entails the representation of one’s own body internal states, achieved by sensing, integrating, and regulating internal body signals [12]. One critical instance is breathing, which provides oxygen and supports olfactory processing, vital mechanisms for human survival. The act of breathing is shaped across mammalians species by the preBotzinger Complex, a medullary microcircuit which elicits the excitation of the descending phrenic nerve, responsible for the lungs and diaphragm muscular contraction [13]. In addition to efferent mechanisms, breathing entails three afferent routes converging to the brain, namely the olfactory, somatosensory and interoceptive pathways, which are respectively mediated by the olfactory bulb, somatosensory and vagal nerves [14].

These anatomical links seem to bear modulatory effects on higher-level brain functions. For instance, nasal breathing has been shown to entrain limbic structures and modulate memory and emotional tasks [15]. The rhythmic contraction of the chest has been shown to cyclically modulate the cortico-muscular synchronization, which is an established long-range communication in the beta range frequency between the sensorimotor cortex and the peripheral muscular contraction [16]. Moreover, a seminal study on mice has demonstrated that a subpopulation of neurons within the respiratory complex is responsible for noradrenaline release via stimulation of the locus coeruleus [17], thereby stressing the causal mechanism of the breath cycle on arousal. Given these diverse links to the central nervous system, it is not surprising that breathing has been shown to have direct relations with perception, emotion, attention and action [14,18–21].

Interoception has also been shown to influence numerical cognition, specifically using a paradigm that requires participants to produce random numbers within a given range. Participants performing the Random Number Generation (RNG) tend to diverge from the rule of randomness, with a systematic bias towards small numbers [22]. Moreover, it has been shown that RNG is also biased by bodily changes, such as head turns [23], eye movements [24] and breathing phase [25]. Belli and collaborators [25] have recently shown that participants produce and perceive larger quantities during inhalation compared to exhalation. Their study entailed two experiments, the first one on the production of random numbers (i.e., RNG) while the second one on the perception of dots numerosity. In the RNG experiment they recruited 22 adults who were instructed to self-monitor their breathing phase and to provide a random number vocally at the end of each exhalation and inhalation phase. The authors observed larger produced numbers during inhalation compared to exhalation with strong effect sizes (𝜼^2^_p_ = ~0.5). Given the positive results, the authors advocated an extension of the ATOM proposal to encompass also interoceptive signals related to breathing and framed them as situated factors in the hierarchical view of cognition proposed by Fischer [8]. Several mechanisms of actions have been discussed, ranging from afferent vagal nerve stimulation to postural and proprioceptive changes that influence concept availability (e.g., “more is up”).

These mechanisms appear to be promising accounts for the understanding of the relation between physiology and cognition. However, there are three major methodological choices which might have impacted the validity of the Belli et al. ‘s conclusion, which were partly discussed by the authors in the study’s limitation section. First, conscious monitoring of the breathing phase and the related linguistic association (e.g., “inhaling is more”) may have artificially induced or increased the relation between breathing and magnitude processing. Second, by requiring responses at the end of each phase, the study ignored the continuous and cyclic nature of the lungs and diaphragm expansion which accompanies respiration. Third, vocal responses interrupt and alter the natural breathing flow. Thus, this study aims to consolidate the hypothesized covariance of natural (“spontaneous”) breathing and numerical magnitude, assessed by means of the RNG, with a novel methodology that addresses the discussed drawbacks.

In this study we probe participants during a RNG based on digit handwriting, while their spontaneous breath is recorded through a respiration belt. Participants give self-paced responses to avoid the engendering of conscious breathing control for action coupling. This paradigm avoids oral responses and considers the continuous nature of respiration, thereby overcoming the main limitations of the study by Belli and colleagues [25]. Moreover, our paradigm allows investigating the effects of breathing on cognition at different levels, by measuring parameters of spontaneous breathing, such as a fine-grained breathing phase and breathing amplitude, and their effects on the cognitive task. Finally, our paradigm allows also investigating the effects of breathing on action, by investigating such effects on the amplitude of writing.

**Fig 1:**
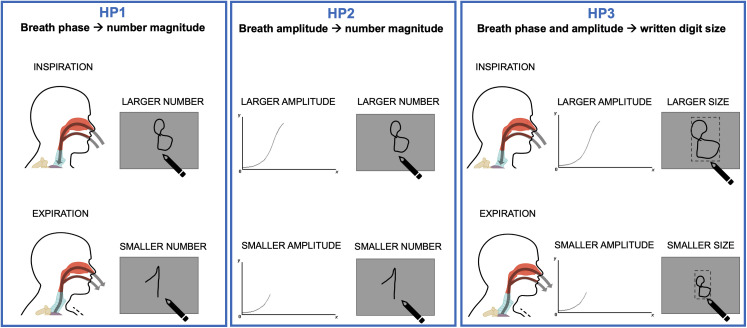
Study hypotheses. The three panels show the three HP of this study. The first HP predicts that breathing relates to the magnitude of the number produced, with larger numbers during inspiration. The second HP states that also the breath amplitude positively correlates with the number produced. The third HP indicates that breathing phase (inspiration) and amplitude relates positively with the size of the written digit.

As a main effect, we expect that the inhalation phase and exhalation phase predict respectively the production of numerically larger and smaller numbers (Hp1); and this will provide a conceptual replication and extension of the results by Belli et al. [25], indicating an effect of spontaneous respiration on the RNG task. As secondary effects, we expect that generated numbers are larger as a function of abdomen expansion, independently of the breathing phase (Hp2). These predicted effects are based on the argument that somatosensory processing due to breathing-related muscular changes interact with high order concepts of magnitudes, inducing associations between smaller/larger number magnitude and smaller/larger amplitude of respiration [25]. Finally, to additionally investigate the effects of breathing on action, we will explore whether breathing phase and amplitude will affect the magnitude of the writing action, with larger or smaller overall written digit size, in terms of width and height, during inhalation or exhalation, respectively (Hp3). The three hypotheses are illustrated in Fig 1. Overall, these hypotheses are all consistent with the proposal that numerical and physical magnitudes also interact with body states.

## Materials and methods

### Sampling plan

Based on the findings from Belli et al. [25], a power analysis was conducted to determine the necessary sample size for this study. In the original study the reported effect size for the influence of the breathing phase on the mean generated number was substantial (partial eta squared, η^2^_p_ = 0.48, equivalent to Cohen’s d = 0.94; [25]). Given the methodological concerns highlighted earlier and the concurrent testing of novel hypotheses (Hp2 and Hp3), the power was set at 99.9% with a significance level (alpha) of 0.05. The estimated sample size required to replicate the same pattern of analyses of the original study consists of 30 participants. Therefore, we plan to test a minimum of 30 participants, with an equal distribution of female and male participants. Importantly, the planned sample size is even larger than that of Belli et al. [25]. Exclusion criteria include prior history of psychiatric or neurological conditions, age below 18 y/o and above 40 y/o, prior history of developmental disorders, use of drugs which might alter cognitive functioning. The study was approved by the local ethical committee (protocol number 837-b).

### Design plan

**Fig 2:**
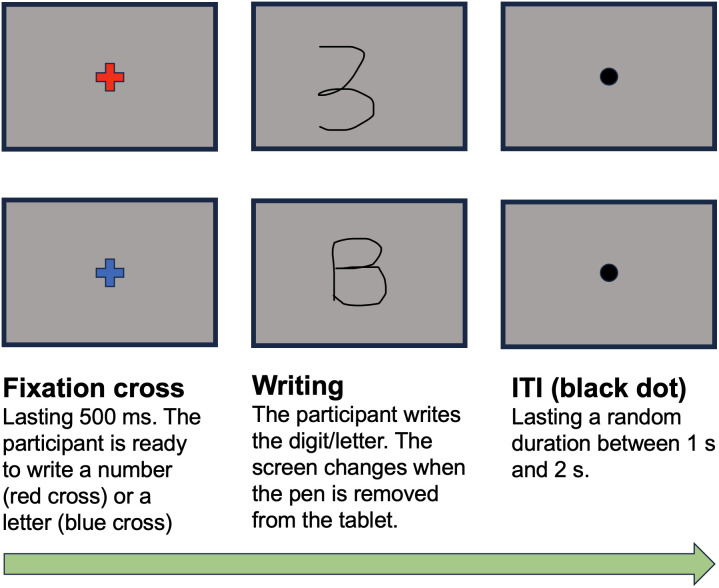
Study paradigm. The frames depict the three main stages implicated in a random number generation trial (bottom sequence) and random letter generation trial (top sequence), with digit/letter writing preceded by a colored central fixation cross indicating the condition (e.g., blue = number, red = letter) and followed by an inter-trial-interval (ITI) signaled by a central black dot.

Participants will first read and confirm a written informed consent form. After providing consent, they will be seated comfortably on a chair in a dimly lit room. The experimenter will position at the level of the abdomen (8^th^ – 10^th^ rib) of the participant a respiratory belt (Piezo-Electric Respiration Sensor, PLUX Biosignals) which detects the abdomen dilation via a force sensor. The experimenter will carefully check the position of the belt and its output signal to ensure data recording quality. A drawing tablet (Wacom) will be placed in front of the participant, with a digital pen for writing, a keyboard, and desktop monitor. After the preparation and instructions of the task, the participant will go through a familiarization phase with 10 trials. The experiment consists of 120 trials total, split equally between 60 trials of RNG and 60 trials of random letter generation (RLG), which serves as a filler condition. Crucially, to discourage advanced planning of the numbers, which would confound the hypothesized link between breathing and RNG, participants will be instructed on both tasks under the pretense that they are of equal importance, keeping them naive to the fact that RNG is the sole condition of interest. Specifically, the participant will have to randomly choose and write down numbers ranging from 1 to 9 for the RNG task or letters from A to Z for the RLG condition. To explain the concept of randomness the same metaphor as suggested by Belli and collaborators [25] for the RNG task will be given: “imagine picking up a number out of a hat, returning it, put it back in the hat, shaking the hat’s contents, then picking another number out of the hat, and so forth”. In each trial the participant will write the number/letter down on the drawing tablet with one continuous stroke. Before starting the experiment, participants will be trained to write digits and letters with one single stroke (particularly 4 and 7, which might be written using two strokes but have simple single-stroke workarounds). To cue the respective tasks, the experiment exploits an informative ‘go’ signal. At the beginning of each trial, a fixation cross colored in blue or red lasting 500 ms will be presented to prompt participants to write down respectively a digit or a letter. Conditions will appear in a random sequence. Noteworthy, to counterbalance the colors assigned to the RLG and RNG conditions, half of the participants will be randomly assigned to the opposite color coding (i.e., red = RNG, blue = RLG). Between each trial there will be a pause, randomly varying according to a uniform distribution between 1000 and 2000 ms accompanied by a central black dot which appears once the participant has finished writing. The timeline of a trial is illustrated in Fig 2. The last trial will be followed by a message indicating that the experiment has terminated. The experiment will run on Open Sesame 4.0 [26]. Scripts to run the experiment are publicly available at the following Open Science Framework repository (https://osf.io/57etu/overview). Once completed data collection and analyses, raw data and analysis scripts will be uploaded in the repository.

### Variables

**Fig 3:**
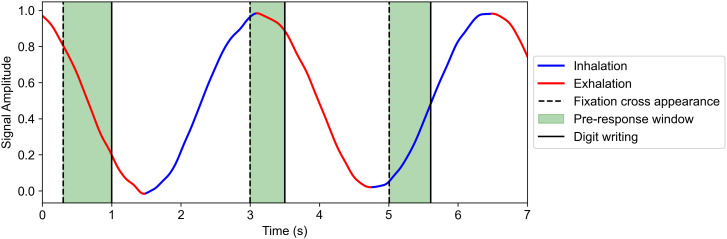
Breathing time series and response event marking. The plot shows a simulated breathing series (normalized signal amplitude of the abdomen expansion) with the ‘Pre-response’ windows highlighted in green which start with the fixation cross appearance, where the mental preparation of the subsequently produced number is assumed to initiate, and finish with the actual start of the digit writing. The respiratory phase (inhalation or exhalation) and magnitude at the start of the ‘Pre-response’ window is hypothesized to influence the cognitive process underlying number generation, biasing for instance towards larger numbers during inspiration.

The written digits will be labeled, and the area covered will be calculated by assessing the height and width of the digit. To account for interindividual differences the area measures will be normalized to a 0–1 unit for everyone separately. The raw signal recorded from the belt, which indicates the abdomen expansion (in volt units), will be preprocessed and then classified into inhalation and exhalation using the NeuroKit2 [27], a toolbox for the analysis of neurophysiological data. Eventually, the preprocessed signal will be normalized to a 0–1 unit for each participant. Assuming participants plan the number right after the start of the trial, the time of response marked in the breathing time series will be at the start of the fixation cross (Fig 3).

Concerning data quality checks, single participant’s data will be discarded, and not replaced, if less than 10% of the responses are marked in either the inhalation or exhalation phases. Moreover, participants with more than 40% of unidentified written digits will be discarded and not replaced.

### Analysis plan

Before testing the main hypotheses of the study results will be analyzed to verify the small number bias and the randomness of the numbers produced. The small number bias effect will be tested with the early-bias index and the random-generation-task-deviation index [1]. The early-bias index corresponds to the total counts of numbers produced below 5 minus the total counts of numbers produced above 5 divided by their sum and multiplied by 100. The random-generation-task-deviation index is the arithmetic mean of the numbers produced by the participants minus the theoretical value (i.e., 5). To verify the small number bias effect the distribution of these indices will be tested against 0 with a one-sample t-test.

To quantify the randomness of the numbers produced we will calculate the random generation index (RGI), as defined by Evans [2]. This index serves as a measure of the conditional redundancy within a sequence, specifically evaluating how often a particular response alternative follows another. The calculation is based on a diagram matrix, a square grid where the dimensions represent the total number of response alternatives (i.e., a 9 X 9 matrix for the numbers 1–9). Each cell (i, j) in this matrix records the frequency with which response j immediately follows response i. By comparing the distribution of these specific response pairs against the total frequency of each individual response, the RGI produces a value bounded between 0 and 1. A value of 0 represents a state of maximum uncertainty or perfect randomness, where all possible response pairs occur with equal frequency. A value of 1 indicates total predictability, where every response in the sequence is perfectly determined by the one preceding it. RGI values will be compared with previously observed findings in the literature.

The main hypotheses of the study are tested with the subsequent two analyses. Type 2 error-rate is set to 0.05.

**HP 1-2.** A Linear Mixed Model (LMM) will be fitted on the produced numbers with breathing phase (categorical variable, inhalation or exhalation) and abdomen expansion (continuous variable) as fixed effects, and within participants random intercepts and random slopes for both fixed effects.

**HP 3.** A LMM will be fitted on the measured areas of the drawn digits. The model will include the breathing phase (categorical variable, inhalation or exhalation) and abdomen expansion (continuous variable), and the number generated as fixed effects, and within participants random intercepts and random slopes for all fixed terms and the number generated. The inclusion as a predictor of the number generated is to control and explore potential linear effects of the number on the magnitude of the drawing.

Importantly, the choice of a LMM is to deal with an imbalanced design, since more responses are expected in the exhalation phase, as suggested by Johannknecht et al. [28] and Park et al. [21] who found a robust bias of the participants in responding during exhalation. In addition, to deepen our understanding on the coupling breathing – magnitude processing, a supplementary analysis is planned which substitutes the simplistic binary predictor ‘inhale vs. exhale’ with four sub-phases of the respiratory cycle (i.e., early vs. late inhalation and early vs. late exhalation). The same HPs and model configuration as outlined above will be held for this supplementary analysis with the exception for the continuous predictor of abdomen expansion which will be dropped to avoid multicollinearity with the four breathing sub-phases categorical predictor. The analysis will be performed upon inspection of the response distribution imbalance in the four sub-phases.
